# TIPS to Treat Chylous Ascites due to Cirrhosis

**DOI:** 10.1002/ccr3.71284

**Published:** 2025-10-14

**Authors:** Muhammad Amir, Asad Ullah Farooq, Rida Zahid, Mahnoor Farooq, Aymar Akilimali

**Affiliations:** ^1^ Integris Health Baptist Medical Center Oklahoma Oklahoma USA; ^2^ King Edward Medical University Lahore Pakistan; ^3^ Rawalpindi Medical College Rawalpindi Pakistan; ^4^ Department of Research Medical Research Circle Goma Democratic Republic of the Congo

**Keywords:** chylous ascites, cirrhotic ascites, hepatic hydrothorax, lymphatic obstruction, portal hypertension, transjugular intrahepatic portosystemic shunt (TIPS)

## Abstract

Chylous ascites is a rare form of ascites that arises from the disruption of lymphatic flow. It develops in patients with liver cirrhosis secondary to portal hypertension. Treatment options include dietary modifications, pharmacological agents, and procedural interventions. Transjugular intrahepatic portosystemic shunt (TIPS) placement has been proposed as a treatment option for chylous ascites in decompensated liver cirrhosis. Our case report discusses a patient with decompensated liver cirrhosis and diuretic‐refractory ascites who ultimately developed chylous ascites that was treated by TIPS placement.

**Summary**

- Chylous ascites is a rare and challenging manifestation of decompensated cirrhosis.
- In patients who are not candidates for liver transplantation, TIPS may provide effective symptom control by reducing portal hypertension.
- It should be considered when conservative management fails and non‐cirrhotic causes have been ruled out.

## 1 Introduction

Chylous ascites is a rare form of ascites that involves the presence of triglyceride‐rich fluid in the peritoneal cavity. It has an incidence reported to be approximately 1 in 20,000 hospital admissions over a 20‐year period. However, the incidence has likely increased due to the longer survival of patients with malignancies and advanced cardio‐thoracic and abdominal surgeries [1].

Chylous ascites develops from disruption of the lymphatic system, which can occur secondary to trauma or obstruction (benign or malignant in etiology). Bhardwaj et al. have classified the underlying mechanism of chylous ascites into portal and non‐portal causes [2]. Two‐thirds of cases in developed countries are attributable to intra‐abdominal malignancies and cirrhosis, whereas infection (e.g., tuberculosis) is the predominant cause in the developing world [1].

The cause of chylous ascites development in cirrhosis is believed to be portal hypertension, though mechanistic details contributing to the development of chylous ascites are somewhat uncertain. It has been hypothesized that elevated portocaval pressures in cirrhosis likely cause increased production of hepatic lymph. Gastrointestinal lymph flow is also increased by splanchnic venous congestion caused by increased portal venous pressure. The excessive lymph flow, which can be up to 20 L/day, can lead to dilation and rupture of serosal lymphatic channels, causing formation of chylous ascites [1, 2, 3].

Because of its rarity in cirrhotic patients, there are no standardized guidelines for treatment. Management of chylous ascites is usually dependent on addressing the underlying etiology. Treatment options include dietary modifications, pharmacological agents, and procedural interventions. In patients with cirrhosis‐related chylous ascites, the condition carries a poor prognosis, with a reported 1‐year mortality rate approaching 60%–70%.

The best treatment option for such patients is liver transplant, but liver transplant is not an option for many patients or may be a premature option for some. For patients who are not transplant candidates or fail conservative management, transjugular intrahepatic portosystemic shunt (TIPS) may provide symptom relief by lowering portal pressure. This case discusses the use of TIPS as a treatment in a patient with cirrhosis who developed chylous ascites.

## 2 Case History/Examination

The patient was a 71‐year‐old female with a history of decompensated cirrhosis due to non‐alcoholic fatty liver disease and alcohol‐associated steatohepatitis. Her cirrhosis was complicated by diuretic‐refractory ascites and diuretic‐responsive peripheral edema. Additional medical comorbidities included hypertension, diabetes mellitus, dyslipidemia, and osteoporosis.

Cirrhosis was initially diagnosed based on imaging performed after she presented with abdominal distension and pedal edema. She was subsequently referred to our hepatology service, where she was started on furosemide, spironolactone, and advised to follow a low‐sodium diet. Initially, therapeutic paracenteses were performed as needed for symptomatic relief of ascites. However, over time, she required increasingly frequent paracenteses—almost weekly—as her ascites became refractory to diuretics.

During this period, the paracentesis fluid developed a milky, white‐colored appearance, and ascitic fluid analysis (Table 1) revealed elevated triglyceride levels consistent with chylous ascites.

**TABLE 1:** Ascitic fluid composition.

Ascitic fluid	
Albumin	0.6 g/dL
WBC	53 cells/μL
RBC	393 cells/μL
Protein	2.3 g/dL
Triglyceride	1009 mg/dL

## 3 Differential Diagnosis, Investigations and Treatment

Differential diagnosis included malignancy, infection (tuberculosis), cardiac causes, and lymphatic obstruction. Investigations, including magnetic resonance imaging (MRI), ascitic fluid cytology, and TB testing, ruled out secondary causes.

MRI of the abdomen ruled out vascular obstruction, peritoneal carcinomatosis, and hepatocellular carcinoma. Additionally, she showed no evidence of constrictive pericarditis or congestive heart failure on cardiologic evaluation. Ascitic fluid cytology and QuantiFERON‐TB Gold test were both negative. Based on the exclusion of non‐portal causes and her known cirrhosis, portal hypertension was considered the most likely underlying mechanism for chylous ascites.

Given her advanced age, poor functional status, and social circumstances, she was not considered a suitable candidate for liver transplantation. Similarly, bowel rest with total parenteral nutrition (TPN) or dietary fat restriction with medium‐chain triglyceride (MCT) supplementation was not feasible.

A lymphangiogram was performed and showed no discrete lymphatic leak amenable to intervention. It was presumed that the patient had microscopic lymphatic oozing due to elevated portal pressures. Her course was further complicated by hepatic hydrothorax.

To improve her quality of life and control the complications of portal hypertension, a TIPS procedure was pursued. A portosystemic shunt was created between the right hepatic vein and the posterior right portal vein using a 7 cm covered stent. Post‐procedure, the porto‐systemic gradient was reduced to 7 mmHg.

In the 6 weeks following TIPS, the patient required three thoracenteses with progressively decreasing fluid volumes. On the fourth attempt, no pleural effusion was found. Notably, she did not require any further paracentesis following TIPS. She was managed on periodic low‐dose diuretics for mild anasarca.

TIPS was thus effective in resolving her chylous ascites and hepatic hydrothorax by reducing portal hypertension. Unfortunately, the patient passed away at home 3 months later due to an unclear cause.

## 4 Conclusion and Results

Following TIPS placement, the patient's ascites and hepatic hydrothorax resolved, confirming effective decompression of portal hypertension.

Overall, there is limited data available to suggest a clear, evidence‐based treatment plan for chylous ascites in cirrhosis patients. Based on our experience, TIPS appears to be a reasonable and effective treatment option, at least in the short term, for the management of chylous ascites in carefully selected patients after non‐portal causes have been excluded and when other strategies are either ineffective or impractical. Further studies are needed to assess the efficacy, durability, and safety of TIPS in larger patient cohorts before it can be recommended as a formal treatment option for cirrhosis‐related chylous ascites.

## 5 Discussion

Chylous ascites can be a nutritionally compromising and difficult‐to‐manage condition. The underlying cause of chylous ascites should be treated whenever feasible. Cases due to infectious, inflammatory, or hemodynamic etiologies may resolve with appropriate therapy. When portal hypertension is the main etiology, however, chylous ascites become especially challenging to manage, as there is usually no focal lymphatic leak to embolize, and treatment of portal hypertension is not always feasible.

There are no formal guidelines regarding the optimal treatment of chylous ascites [3]. A stepwise approach, progressing from conservative management to invasive procedures, is generally recommended [4]. Dietary modification with a high‐protein, low‐fat diet supplemented with MCTs is initially advised, though this may not be feasible in many patients due to social or economic constraints—as was the case for our patient [1, 2, 5, 6]. Moreover, the benefit of MCTs in advanced cirrhosis is equivocal; some studies have reported neurotoxicity and worsening hepatic encephalopathy from elevated MCT levels [2].

Patients with cirrhosis‐associated chylous ascites can be partially managed with sodium restriction and diuretics, although the response is often incomplete [1]. If these strategies fail, bowel rest with TPN can be implemented to reduce lymphatic flow [1, 2, 6]. However, this approach may be impractical in most cirrhotic patients, who are immunocompromised and at elevated risk for infections. Pharmacological agents such as orlistat and octreotide have been used in select cases, particularly in conjunction with dietary interventions [2, 6, 7].

Symptom relief through therapeutic paracentesis is possible but requires frequent procedures, which can lead to complications such as prolonged fluid leakage, spontaneous bacterial peritonitis, bleeding, frailty, renal impairment, and nutritional deficiencies [1, 6, 8, 9].

Development of portal hypertension in cirrhosis ultimately leads to ascites. Therefore, addressing portal pressure is key to managing chylous ascites. TIPS reduces portal hypertension by creating a shunt between the portal and hepatic veins, thereby decompressing the mesenteric venous system. This, in turn, leads to a reduction in lymphatic flow and pressure [2].

Although TIPS has theoretical benefits in treating cirrhosis‐associated chylous ascites, reported experience is limited. Chylous ascites constitutes only 0.5%–1% of all cirrhosis‐related ascites cases [3, 6, 10]. A 2013 literature review by Rajesh et al. identified just seven case reports and one case series (involving four patients) where TIPS was used for this indication [10]. A separate 2016 analysis by Tsauo et al. found that the mean portosystemic gradient decreased from 19.9 to 8.1 mmHg following TIPS in such patients [3].

To contextualize our case, we summarize previously published reports of TIPS use for chylous ascites in Table 2.

**TABLE 2:** Previously reported cases of TIPS for treatment of chylous ascites.

Study	Year	Underlying cause	No. of patients	TIPS outcome	Notes
Rajesh et al. [10]	2013	Cirrhosis (varied etiologies)	7 cases + 1 series (4 pts)	Symptom resolution in most	1 patient required shunt revision
Tsauo et al. [3]	2016	Cirrhosis/chylothorax/chylous ascites	6	↓ PSG from 19.9 → 8.1 mmHg	Included chylothorax cases
Other case reports [4, 5]	Various	Cirrhosis	Individual reports	Resolution or marked improvement	Limited follow‐up data

Other invasive approaches such as peritoneovenous shunting have been attempted to redirect chylous fluid into the systemic circulation. However, this technique is associated with a high risk of sepsis, disseminated intravascular coagulation, hypokalemia, small bowel obstruction, and air embolism, limiting its use [2, 6].

Surgical ligation or percutaneous embolization may be considered in cases with identifiable lymphatic leaks refractory to medical therapy [2, 3, 6]. However, these approaches do not address the underlying portal hypertension in cirrhotic patients. In addition, surgical interventions carry substantial risk of decompensation in patients with advanced liver disease, making them poor candidates for abdominal surgery [3, 11].

## Author Contributions

**Muhammad Amir:** conceptualization, resources. **Asad Ullah Farooq:** conceptualization, writing – original draft. **Rida Zahid:** writing – review and editing. **Mahnoor Farooq:** writing – original draft. **Aymar Akilimali:** writing – review and editing.

## Consent

Written informed consent for publication of this clinical case report, including the use of any associated images and medical data, was obtained from the patient prior to submission. The patient's identity has been anonymized to maintain confidentiality.

## Conflicts of Interest

The authors declare no conflicts of interest.

## Data Availability

The authors have nothing to report.
